# Molecular Mechanisms of Muscle Tone Impairment under Conditions of Real and Simulated Space Flight

**DOI:** 10.32607/actanaturae.10953

**Published:** 2021

**Authors:** B. S. Shenkman, A. K. Tsaturyan, I. M. Vikhlyantsev, I. B. Kozlovskaya, A. I. Grigoriev

**Affiliations:** State Scientific Center of Russian Federation – Institute of Biomedical Problems, Moscow, 123007 Russia; Lomonosov Moscow State University Research Institute of Mechanics, Moscow, 119192 Russia; Institute of Experimental and Theoretical Biophysics, Moscow Region, Pushchino, 142290 Russia

**Keywords:** skeletal muscle, gravitational unloading, atonia, hindlimb suspension, dry immersion, muscle stiffness, intrinsic stiffness, passive stiffness, cytoskeleton, sarcomeric cytoskeletal proteins, titin, collagen, signaling

## Abstract

Kozlovskaya *et al. *[1] and Grigoriev *et al. *[2] showed that enormous loss of muscle stiffness (atonia) develops in humans under true (space flight) and simulated microgravity conditions as early as after the first days of exposure. This phenomenon is attributed to the inactivation of slow motor units and called reflectory atonia. However, a lot of evidence indicating that even isolated muscle or a single fiber possesses substantial stiffness was published at the end of the 20th century. This intrinsic stiffness is determined by the active component, i.e. the ability to form actin-myosin cross-bridges during muscle stretch and contraction, as well as by cytoskeletal and extracellular matrix proteins, capable of resisting muscle stretch. The main facts on intrinsic muscle stiffness under conditions of gravitational unloading are considered in this review. The data obtained in studies of humans under dry immersion and rodent hindlimb suspension is analyzed. The results and hypotheses regarding reduced probability of cross-bridge formation in an atrophying muscle due to increased interfilament spacing are described. The evidence of cytoskeletal protein (titin, nebulin, etc.) degradation during gravitational unloading is also discussed. The possible mechanisms underlying structural changes in skeletal muscle collagen and its role in reducing intrinsic muscle stiffness are presented. The molecular mechanisms of changes in intrinsic stiffness during space flight and simulated microgravity are reviewed.

## INTRODUCTION

The mysterious mechanisms of maintaining and decreasing muscle tonus have always attracted the attention of physiologists. The tone is usually referred to as mechanical tension in the relaxed muscle, which provides a biomechanical basis for performing directed movements. A change in the tone can be assessed by changes in muscle stiffness. Reflex control of muscle tone has been known for a long time. Whether the muscle possesses molecular and cellular mechanisms to maintain its tone still remains a controversial issue.

**Fig. 1:**
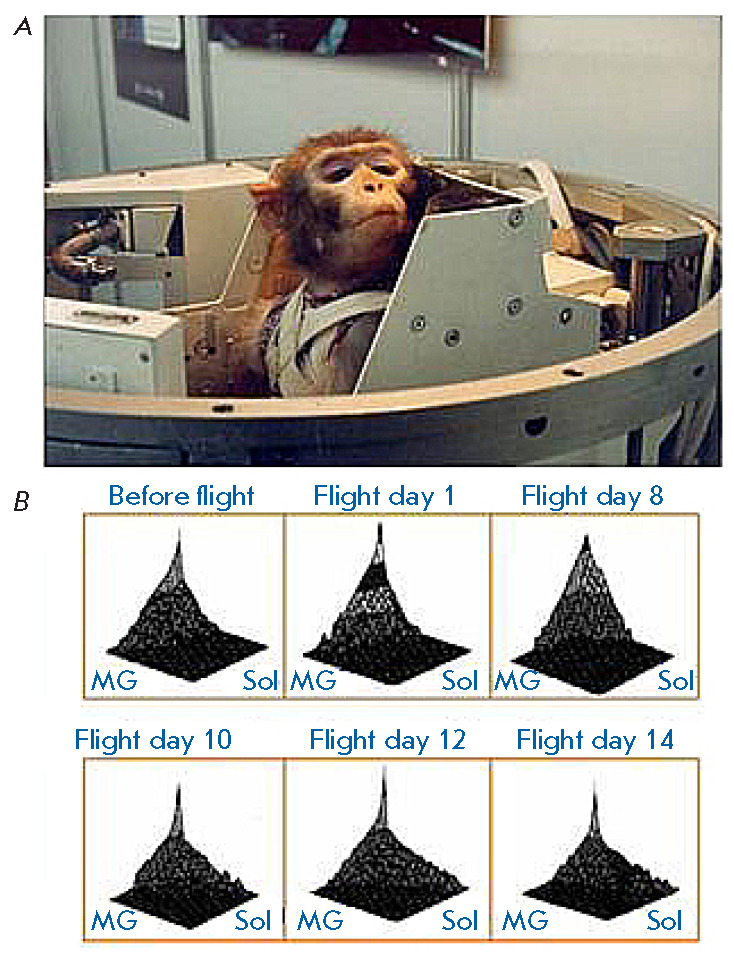
Changes in the recruitment order of the rhesus monkey gastrocnemius and soleus muscles in a foot lever pressing task with sustained load during space flight aboard a biosatellite. Slow-twitch fiber comprise up to 95% of the soleus muscle. The percentage of slowtwitch fiber in the gastrocnemius muscle does not exceed 40–50%, the rest of the fibers are fast-twitch ones. Monitoring of the EMG activity of these two muscles during a lever-pressing task in the biosatellite capsule showed that the movement was performed mainly by the soleus muscle before flight. The pattern changed from day to day during the space flight: soleus activity decreased, while gastrocnemius activity increased. Thus, the task was performed almost completely by the gastrocnemius muscle by the end of the 2-week flight

**Fig. 2:**
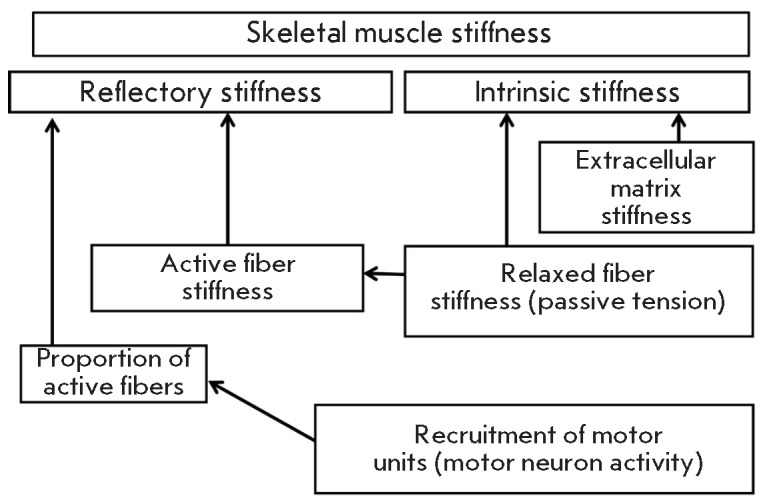
Physiological classification of skeletal muscle stiffness characteristics

Kozlovskaya *et al. *[1] and Grigoriev *et al. *[2] used tensometric and vibrometric methods to assess transverse stiffness in human muscles *in vivo *and observed a significant loss of muscle stiffness as early as in the first days of exposure under both true (space flight) and simulated microgravity conditions. This phenomenon is called hypogravity-induced atonia. The loss of stiffness is associated mainly with changes in the performance of extensor motor units: i.e., inactivation of a pool of slow-twitch motor units during gravitational unloading [3, 4] (*Fig. 1*). These concepts are supported by several observations indicating a significant decrease or complete cessation of electrical activity in the rat postural soleus muscle under support withdrawal during both ground-based experiments with hindlimb suspension and real microgravity created by Kepler orbit flight [5, 6, 7, 8]. Therefore, we suggest that stiffness is lost largely due to the inactivation of the slow muscle fibers that maintain baseline mechanical activity in the muscle even at rest on Earth, which, in turn, influences muscle stiffness parameters *in vivo*. This stiffness component may be called reflectory stiffness (*Fig. 2*).

Are there any intrinsic peripheral mechanisms for reducing muscle stiffness during its inactivation?

**Fig. 3:**
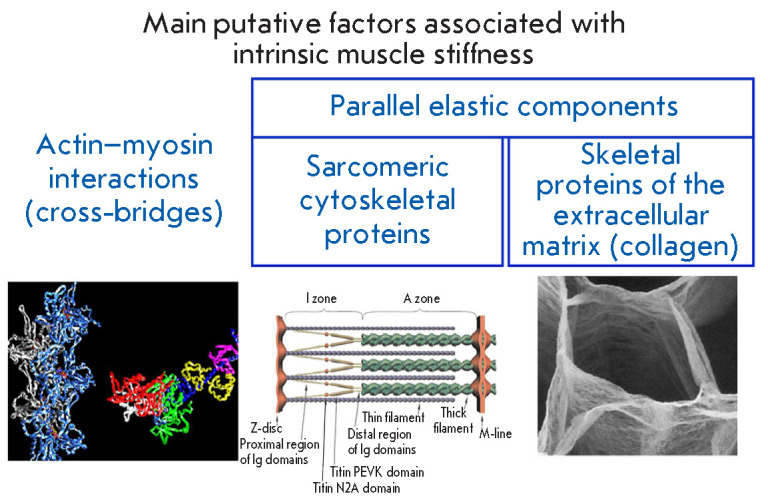
Main putative factors associated with intrinsic muscle stiffness

By the end of the 20th century, there was a lot of evidence indicating that even an isolated muscle or an isolated (and permeabilized) fiber has functionally significant stiffness that is gradually lost after cessation of contractile activity. This intrinsic muscle stiffness (*Fig. 2*) is controlled by both the active component, i.e. the ability to form some of the actin-myosin bonds (cross-bridges) during stretching and contraction, and the parallel elastic component, i.e. structural proteins of the cytoskeleton and extracellular matrix, which are capable of exerting mechanical resistance during muscle/fiber stretch and contraction (*Fig. 3*).

Stiffness is an increase in the mechanical tension, i.e. the tensile force per cross-sectional area (CSA), in response to deformation (relative elongation) of muscle fibers. Since a muscle cell, especially one that is activated, exhibits not only elastic, but also viscoelastic properties, the result of determining the stiffness depends on the method of measurement used. There is dynamic or instantaneous stiffness, which can be measured by applying a very rapid deformation, and static stiffness, which is characterized by the level of tension established long after the end of length change. There are stepwise (rectangular), sawtooth or sinusoidal patterns of muscle length changes used for stiffness measurements. In the first case, the muscle is subjected to step length changes lasting about 0.1 ms in the best experimental conditions, which enables measuring of instantaneous stiffness. In the second case, the muscle length is changed linearly, which enables direct measurement of the length-tension curve during loading or unloading. Sinusoidal or harmonic stretching allows for the best use of available equipment in order to achieve maximum time resolution. Due to the nonlinearity of the muscle stress-strain diagram in response to as small as a few percents stretching, the tangent and secant or chordal stiffness types are different. Active stiffness of an intact muscle can be caused by background electrical potential, and that of an isolated muscle is associated either with the presence of a suprathreshold concentration of calcium ions causing partial activation of the troponin–tropomyosin regulatory system or with defects in this system: e.g., partial loss of troponin complexes resulting in activation of some regulatory units even in the absence of calcium ions. The active stiffness component can be eliminated by adding blebbistatin, a specific myosin II inhibitor that penetrates the cell through the sarcolemma [9], binds myosin, and inhibits its transition to the strong actin-myosin complex [10]. The active stiffness component can be precisely measured by applying sufficiently rapid stretching, with deformation rates of at least several muscle lengths per second. Otherwise, the stiffness value is underestimated due to stress relaxation. Since passive stiffness is nonlinear, the entire length-tension curve (tensile force per CSA) should be recorded.

**Fig. 4:**
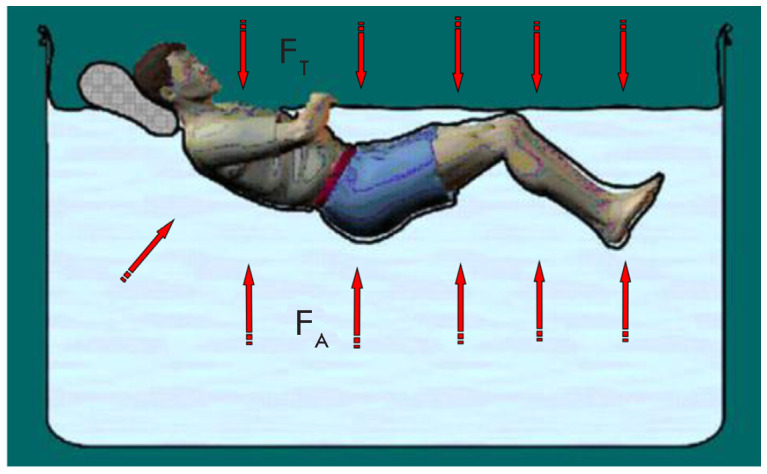
An experimental model of dry immersion. When the body is immersed in water, the resulting force of hydrostatic pressure (Archimedean force, FA) balances the force of gravity (FT). However, the Archimedean force is distributed over the entire body surface. Because of that, the pressure on each point per unit body surface area is much lower than the support reaction force in the contact area in an upright, sitting, or prone position

**Fig. 5:**
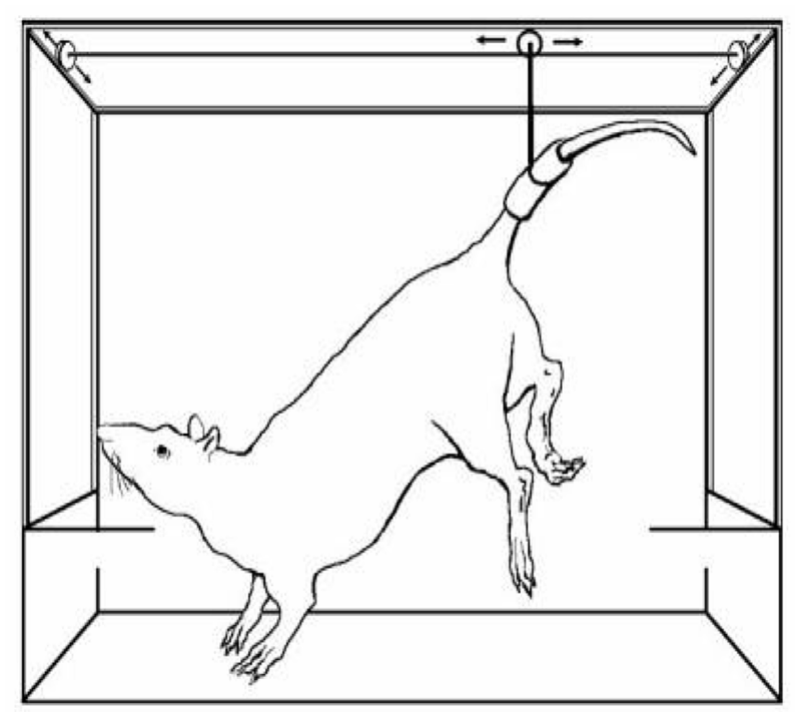
An experimental simulation model of rodent hindlimb suspension. After detachment of the animal’s foot from the ground support, afferents are activated and the animal turns out to be under unloading conditions

This review discusses the central data on the changes in intrinsic muscle stiffness under conditions of gravitational unloading that mainly result in deep inactivation of many muscles. We will primarily analyze the data obtained under support withdrawal conditions, i.e. in experiments using a dry immersion model (with the participation of volunteers, *Fig. 4*) and, then, hindlimb suspension (using laboratory rodents, *Fig. 5*). We will also discuss the putative mechanisms of a decline in intrinsic muscle stiffness and the role of this decline in muscle atrophy.

Prior to discussing the issue at hand, we would like to briefly describe the experimental approaches mentioned above.

Dry immersion is a model developed in Russia in the 1970s [11]. It involves complete water immersion of the subject in an open bath. The subject’s body surface is separated from the water by a waterproof piece of fabric covering the water surface and bath edges, with the subject head only exposed to air (*Fig. 4*).

Hindlimb suspension [12, 13] remains one of the most commonly used microgravity models in laboratory rodents. The animal is suspended below the cage ceiling either by the tail, back skin, or a cloth vest so that the forelimbs rest on the ground, while the hindlimbs hang at an angle of 30–40 degrees to the floor (*Fig. 5*). If the model is used correctly, the animal can move freely inside the cage. The level of corticosterone indicating the degree of animal stress rarely exceeds that of an intact control rodent [14].

## PASSIVE AND ACTIVE STIFFNESS OF ISOLATED MUSCLE AND FIBER DURING GRAVITATIONAL UNLOADING

**Fig. 6:**
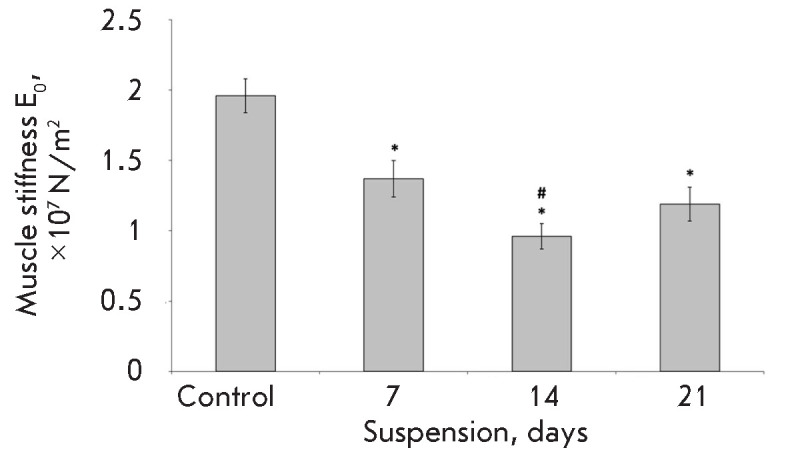
Changes in the dynamic elastic Young’s modulus (stiffness index) of fully activated permeabilized fiber of the rat soleus muscle during hindlimb suspension [17] after 7, 14, and 21 days. * –significant difference from the control group (*p* < 0.05), # –significant difference from the 7-day unloading group (*p * < 0.05)

Gravitational unloading is known to decrease significantly both the passive and active stiffness of muscle and muscle fiber. Goubel *et al. *demonstrated that passive tension of the rat postural soleus muscle significantly reduces after 3–4 weeks of suspension [15]. As early as in their first work, the authors attributed a decline in the series elastic component to both the active mechanisms (cross-bridges) and the passive (in the authors’ opinion, mainly tendon) elements. However, a decline in the passive tension was also established in single permeabilized soleus muscle fiber after 14-day suspension [16]. Furthermore, as shown in an experiment with elimination of the effect of actin- myosin bonds, this decline may be, for the most part, associated with a decrease in the relative content of titin, an elastic cytoskeletal protein. The time course of the changes in the dynamic stiffness of fully activated muscle fibers under simulated gravitational unloading (suspension) was investigated by McDonald and Fitts [17]. The Young’s modulus decreased by 30% after seven days of unloading and by 50% after two weeks of suspension compared to that in the control animals (*Fig. 6*). Interestingly, the modulus value after three-week suspension remained the same as after two weeks of unloading. Transverse stiffness of permeabilized soleus muscle fiber in suspended rats was evaluated by atomic force microscopy in the laboratory of one of the authors of the current review. An analysis of the contractile apparatus with this method, following detergent-based removal of membrane structures, revealed that transverse stiffness of the myofibrillar apparatus in the area from the M-line to the Z-disc was statistically significantly reduced by 35% only on the third (but not on the first) day of suspension. The stiffness then decreased slower, but transverse stiffness was 68% lower than in the controls by day 12 of suspension [18]. Transverse stiffness in the Z-disc region dropped more than two-fold by day three of suspension and further continued to decrease. Interestingly, measuring the transverse stiffness of the contractile structures of a muscle fiber activated by a high concentration of Ca^2+^ ions (pCa 4.2) revealed a much more pronounced decline in the stiffness after suspension: an almost two-fold reduction in the region between the Z-disc and the M-line after three days and a more than 63% decrease after 12 days. It should be noted that, since activated fiber stiffness was almost two-fold higher than that of relaxed fiber in an intact animal, the absolute value of a decline in activated fiber stiffness was significantly higher. Similar data were obtained for the human soleus muscle in an experiment with volunteers after seven days of dry immersion [19]. When considering these data, one has to take into account the limited capabilities of atomic force microscopy: the inability to capture the longitudinal resistance of a sample, as well the stiffness of the whole fiber/muscle due to the limited depth of cantilever penetration.

Thus, the data available to date do not question the decline in intrinsic longitudinal and transverse, dynamic and static, as well as passive and active, stiffness of the muscle, its fibers, and their components upon simulated gravitational unloading of mammals. However, the molecular mechanisms underlying this decline in stiffness remain unclear.

## MOLECULAR FACTORS AFFECTING MUSCLE STIFFNESS: CROSS-BRIDGES

Cross-bridges [20 , 21, 22], as well as cytoskeletal (titin, nebulin, obscurin, and myosin-binding protein C) and regulatory proteins, determine passive muscle stiffness during stretching. These proteins constitute the passive parallel elastic component of the muscle [23, 24] and affect the probability of cross-bridge formation [25, 26, 27, 28].

Interfibrillar matrix components, in particular collagen fibrils, also determine the stiffness of the entire muscle or its fiber bundles [29]. Extracellular matrix stiffness was recently shown to be significantly higher than that of isolated fiber [30]. Studying the effect of gravitational unloading on these proteins is of great interest. Passive stiffness is higher in muscle predominantly composed of fiber expressing slow myosin heavy chains [15]. Therefore, one would expect that stiffness should decrease under gravitational unloading due to a change in the expression pattern of myosin heavy chain isoforms in favor of fast-twitch isoforms, provided that all the other parameters are equal [31, 32].

The probability of cross-bridge formation is higher if the interfilament spacing in the myofibrillar apparatus is optimal. A decrease in the relative number of normally arranged actin filaments (in the absence of structural disturbances) should increase the interfilament spacing and should reduce the probability of cross-bridge formation. Fitts and Riley noted a reduced amount of actin filaments and shortening of some of them in the soleus muscle after 14 days of suspension in rats [33], 17 days of bed rest, and 17 days of space flight [34, 35, 36]. These changes are accompanied by a decrease in the maximum force and power of contraction of single permeabilized fibers, as well as in their calcium sensitivity. The discovered phenomenon may be directly associated with reduced active muscle stiffness. The cause of these changes has not yet been established. Previously, we noted a decrease in the content of nebulin, a thin filament protein, in the rat soleus muscle after 7–14 days of suspension [37, 38]. A possible cause of the “loss” of actin filaments may be a decrease in the relative nebulin content. Meanwhile, it has recently been established that the number of strong actin–yosin bonds in a genetically atrophied muscle decreases, while the number of weak actin–yosin bonds in the muscle increases during isometric contraction (based on EPR data) [39]. In an experiment with hindlimb suspension in rats, we have recently shown that the specific and effective inhibitor of myosin II blebbistatin has the same effect on passive stiffness of the soleus muscle in both an intact animal and an animal with reduced passive stiffness, after three days of gravitational unloading. These results suggest that a possible change in the parameters of a small number of the cross-bridges formed in a resting muscle after gravitational unloading does not affect its passive stiffness [40]. However, one cannot exclude the possibility that increasing interfilament spacing, decreasing the number of thin filaments, and changing the parameters of cross-bridges in unloading and hypogravity-induced atrophy may significantly affect active dynamic stiffness. This issue is a challenge for future research.

## SARCOMERIC PROTEINS AND MUSCLE STIFFNESS

**Fig. 7:**
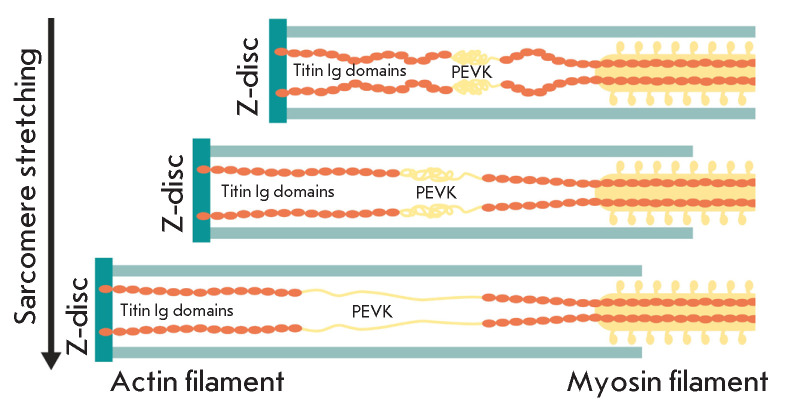
Spring-like properties (compliance) of the titin PEVK domain

Among sarcomeric cytoskeletal proteins, titin attracts the most attention; its contribution to passive muscle stiffness is considered to be very significant [23, 41]. Several domains of a giant titin molecule have, to greater or lesser extent, spring-like properties and can compress and stretch (*Fig. 7*). A decrease in the relative content of titin during hindlimb unloading was first discovered by Christine Kasper in 2000 [42]. Similar data were obtained in a laboratory of the University of Lille in 2002 [16]. In the same year, we found a decrease in the level of titin-1 (T1) and an increase in the level of its proteolytic fragment T2 in the rat soleus muscle after 14-day hindlimb unloading [43]. Given that titin is one of the constituents of the parallel elastic component determining the value of fiber passive stiffness that reduces during unloading, one might expect either a decrease in the content of this protein or an increase in its compliance as early as 2–3 days after hindlimb unloading (when passive muscle stiffness is already decreased). However, this turned out to be not entirely true. Goto *et al. *found no changes in the connectin (titin-1) content after three days of hindlimb unloading [44]. In this case, an elastic region of the titin molecule that is located between the Z-disk and the N2A-domain (including PEVK spring region) was found to lose its elasticity instead of increasing it, thus showing less elastic properties after hindlimb unloading [44]. These data have recently been explained in a study by Nishikawa *et al. *[45], who demonstrated that an increase in the calcium ion level in a fiber (which takes place during gravitational unloading [46, 47, 48]) results in rigid binding of a titin molecule to thin filaments in the N2A domain. In 2008, we also found no decrease in the content of a N2A titin-1 isoform, typical of skeletal muscles, in the rat soleus muscle after three days of hindlimb unloading [49]. A significant decrease in the titin-1 content was noted after seven days of hindlimb unloading [50]. A statistically significant increase in titin expression in the rat soleus muscle during three days of unloading (hindlimb suspension) was recently revealed in the laboratory of one of the authors of this review [51]. It is possible that this increased expression compensates for the breakdown of some titin molecules, which leads to the lack of visible changes in its content. Interestingly, the titin expression level did not exceed the control after seven days of hindlimb unloading [50], which made it possible to register a decrease in the titin content at this time interval, which is probably due to its enhanced calcium-dependent proteolysis.

Thus, there is good evidence to suggest that the destruction of titin and nebulin during exposure of an animal to simulated gravitational unloading for more than three days can contribute significantly to a decline in passive muscle stiffness. However, the question of whether alterations in this protein can be associated with changes in the stiffness properties of an unloaded muscle in the early period of unloading (up to three days) remains open. Likely, a change in the degree of protein phosphorylation may contribute to a change in the stiffness of the titin molecule and, respectively, the entire muscle in the early period of unloading. There are grounds for this suggestion. Phosphorylation/dephosphorylation of PEVK and N2B domains in cardiac muscle titin is known to alter the stiffness properties of the molecules, leading to a change in the titin-based passive stiffness of cardiomyocytes and the entire muscle [52]. These changes, in turn, play an important role in the regulation of myocardium contractile activity. There is evidence of phosphorylation of skeletal muscle titin [53, 54]. The role of this post-translational modification in changing the stiffness properties of the titin molecule is unclear. However, these changes have been suggested to play a role in reducing titin-based passive stiffness, as based on data demonstrating a decreased level of PEVK region phosphorylation in titin in the rat vastus lateralis muscle after 15-minute physical activity (treadmill running) [54]. The role of titin hypophosphorylation in the decrease in the stiffness of its molecules and the compromising of the contractile ability of the rat diaphragm after 18-hour mechanical unloading (mechanical lung ventilation), leading to muscle atrophy, is also discussed [55, 56, 57]. We found an increase in the total T1 and T2 phophosphorylation level resulting in a decreased T1 content in the mouse gastrocnemius muscle after a 30-day space flight [58]. Reduced titin and nebulin contents under gravitational unloading would undoubtedly decrease the passive stiffness developed by titin molecules upon stretching, as well as general muscle stiffness. However, titin stiffness can both decrease and increase. depending on which molecule regions are phosphorylated.

A hypothesis linking the breakdown in some cytoskeletal proteins (presumably affecting muscle stiffness) to phosphorylation of specific sites in their molecules cannot be excluded. This hypothesis has recently been confirmed in studies on the mechanisms of desmin (intermyofibrillar and intermyofilament cytoskeletal protein) breakdown. Cohen *et al. *showed that phosphorylation of desmin by the well-known kinase GSK3β triggers ubiquitination and calpain-mediated depolymerization of desmin [59]. The kinase can be inhibited via negative phosphorylation by kinase Akt1 [60] and NO-dependent kinase of the guanylate cyclase cascade [61]. Thus, phosphorylation/dephosphorylation of desmin can affect both the protein content and the degree of intrinsic muscle stiffness.

The phosphorylation level of myosin light chains, primarily in fast-twitch fiber, is of great importance for cross-bridge formation. Phosphorylation of myosin light chains by light chain kinase promotes crossbridge formation and enhances the calcium sensitivity of permeabilized fiber [62, 63]. However paradoxical it may sound, the phosphorylation level of myosin light chains in the rat soleus muscle increases, and does not decrease, under simulated gravitational unloading (hindlimb suspension model), as it was shown at the beginning of this century [64]. Thus, an elevated phosphorylation level of myosin light chains under gravitational unloading can, to some extent, compensate for a decline in muscle stiffness caused by an increase in intermyofilament spacing, a decrease in the number of thin filaments, and a decrease in the content of the sarcomeric cytoskeleton protein titin.

The myosin-binding protein C plays the most important role in cross-bridge formation. A phosphorylated (at three sites) protein acts as a scaffold in the actin– myosin cross-bridge assembly [65]. However, we failed to find any data describing this protein’s state during unloading. The same can be said for another important sarcomeric protein, obscurin.

Another protein, telethonin, anchors adjacent titin filaments in the Z-disc and, therefore, plays an important role in maintaining the Z-disc structure and integrity, as well as titin cytoskeleton integrity. Taillandier* et al. *showed that hindlimb suspension causes telethonin ubiquitination and breakdown in the rat soleus muscle [66]. Interestingly, the telethonin content decreases significantly after three days of hindlimb unloading [40].

One of the authors of this review found that gravitational unloading leads to a degradation of alpha-actinin- 2, a characteristic Z-disc protein [67]. This degradation becomes statistically significant only after seven days of hindlimb suspension. Interestingly, the content of alpha-actinin-3 in the rat soleus muscle decreases by 20% already after three days of hindlimb unloading [40]. Probably, a decreased content of alpha-actinins-2 and -3 may, to some extent, lead to Z-disc disintegration. This, in turn, may compromise interfilament spacing stability and reduce the chance of cross-bridge formation, which contributes to a decreased active muscle stiffness. It should also be noted that, like telethonin, alpha-actinins anchor titin in the Z-disc [68]. Their destruction can result in disintegration of the entire sarcomeric cytoskeleton and reduced muscle stiffness.

**Collagen**

**Fig. 8:**
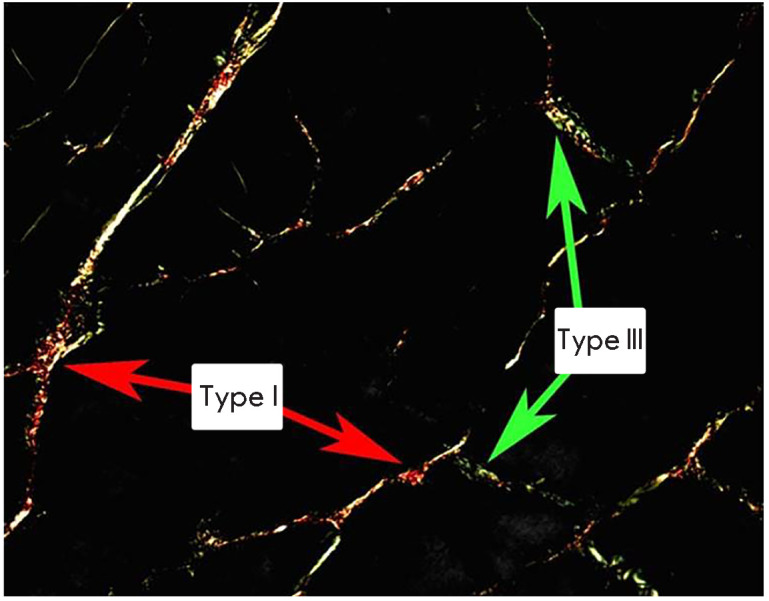
Collagen isoforms: collagen I (red) and collagen III (green). A transverse section of the human soleus muscle is shown. The sample was stained in picrosirius red and examined by polarization microscopy

Passive stiffness of the extracellular matrix and connective tissue of the skeletal muscle is an important component of the whole muscle stiffness. This stiffness significantly exceeds passive stiffness of muscle fiber and exhibits a pronounced nonlinear dependence [30, 69, 70]. The main factor determining the mechanical properties of the extracellular matrix and muscle connective tissue is the number and properties (such as the number of hydroxyproline cross-links) of collagen fibrils. Several different collagen isoforms are present in skeletal muscles. Collagens I and III make the greatest contribution to the muscle’s mechanical properties [71]. Of these, collagen III has lower stiffness and greater elasticity (*Fig. 8*).

The contribution of collagen to passive stiffness of the whole muscle is undeniable. However, it is currently unclear to which extent breakdown and reduced synthesis of collagen during unloading affect a decline in stiffness. Despite a progressive increase in the connective tissue volume under conditions of gravitational unloading [72, 73], no increase in the collagen content was recorded in muscle during these experiments [74]. On the contrary, a significant decrease in the collagen content was observed in the soleus, plantar, and some other hindlimb muscles in rats after a 7-day space flight [75]. Similar data were obtained during immobilization of the soleus muscle in a shortened position [76]. A pronounced decrease in the level of type I and III collagen mRNAs was observed on day three of an experiment simulating gravitational unloading by hindlimb suspension in rats [77]. The collagen mRNA level reached its control level by day seven of the experiment [77]. The expression of collagen III mRNA in the soleus muscle decreases after seven days of hindlimb suspension [78]. At the same time, a significant drop in the expression of all muscle collagen isoforms was revealed mainly in the fast-twitch gastrocnemius muscle after 3-week hindlimb suspension [79]. Analysis of collagen expression in the human vastus lateralis and soleus muscles after 90-day bed rest showed no significant changes [80]. An interesting phenomenon was observed after 14 days of hindlimb unloading: a shift in the expression ratio of type I collagen (a stiffer isoform) and type III collagen (a more elastic isoform) in favor of type III collagen [81]. It is unknown how this phenomenon can affect muscle stiffness. Considering the above, it is clear that the collagen state in a postural muscle under gravitational unloading has not been studied enough yet. Therefore, it is difficult to evaluate the role of collagen types in the decrease in passive muscle stiffness during unloading.

**Molecular mechanisms of reducing intrinsic muscle stiffness**

The available data indicate that intrinsic muscle stiffness is mainly associated with the state of sarcomeric cytoskeletal proteins. In this regard, we are considering here the concepts on the mechanisms of a decrease in inactivated muscle stiffness, based on knowledge on the breakdown of these proteins.

Degradation of a number of cytoskeletal proteins, in particular titin, is known to involve calcium-dependent cysteine proteases: calpains [82]. Murphy *et al. *demonstrated that treatment of a permeabilized fiber specimen with a μ-calpain solution results in a rapid decline in passive force: i.e., stiffness. In addition, rapid proteolysis of titin was observed. The role of calpains during gravitational unloading has been intensively studied in recent years. For instance, calpain activity was shown to significantly increase in the first days of suspension (albeit measured in a lysate in the presence of calcium ions at a supraphysiological concentration), while desmin underwent rapid decomposition [18, 83 , 84, 85]. Interestingly, calpain activation is associated with structural abnormalities in the Z-disc in muscle fiber [86]. We found that prevention of excessive accumulation of calcium ions in muscle fiber using a calcium-binding agent or an inhibitor of dihydropyridine calcium channels (nifedipine) reduces μ-calpain activity [85]. Another interesting finding is that inhibition of calcium channels decreases the level of μ-calpain mRNA, which is elevated under unloading conditions [87].

All these data indicate the high activity of calpain during unloading, which should contribute to rapid breakdown of cytoskeletal and regulatory sarcomeric proteins and decreased muscle stiffness. Indeed, the use of the specific calpain inhibitor PD150606 not only prevented degradation of cytoskeletal proteins that stabilize titin (α-actinin-2 and telethonin), but also reduced passive stiffness of the soleus muscle [40].

Endogenous calpain inhibitors include calpastatin and nitric oxide. Mice overexpressing the calpastatin gene showed no atrophic changes during hindlimb unloading [88]. Calpastatin expression in healthy animals, on the contrary, decreases during hindlimb unloading [84]. Unfortunately, no physiological mechanisms depending on the level of muscle activity and regulating calpastatin expression are known to date. Another endogenous calpain inhibitor is nitric oxide [89]. Its production depends on the muscle contractile activity [90]. The production of nitric oxide decreases during muscle unloading [91]. At the same time, administration of *L*-arginine to increase the level of nitric oxide in an atrophied muscle prevents breakdown of a number of cytoskeletal proteins and, to some extent, reduces the severity of muscle atrophy [91]. We have recently obtained data indicating prevention of titin breakdown during gravitational unloading upon *L*-arginine administration [50]. Thus, we may suggest that a reduced level of nitric oxide during gravitational unloading contributes to decreased muscle stiffness thanks to calpain-mediated breakdown of cytoskeletal proteins.

Another group of factors preventing proteolysis of cytoskeletal proteins is the heat shock proteins (HSPs) that activate neuronal NO synthase and ensure titin integrity [92, 93]. The degradation of contractile proteins can be enhanced by breakdown of Hsp90 and 70 heat shock proteins, which are usually present at very high concentrations in a muscle. However, their level drops by 50–70% during gravitational unloading due to muscle atrophy [94, 95]. Some authors believe that decreased Hsp expression in muscles during unloading may be of significant importance in muscle atrophy. A sharp rise in the level of Hsp90 and Hsp70 proteins was obtained using the 17-AAG inhibitor during gravitational unloading [96]. The Hsp90 inhibitor 17-AAG prevented an increase in the calpain level and intensification of protein ubiquitination. The active Hsp90– neuronal NO synthase interaction and its protective effect on titin suggest that decreased HSP90 expression during gravitational unloading may be associated with reduced muscle stiffness.

Although most authors agree that extracellular matrix proteins, in particular collagen isoforms, significantly contribute to the control of intrinsic passive muscle stiffness, changes in these proteins during unloading have been studied much less than changes in sarcomeric cytoskeletal proteins. Thus, investigation of the mechanisms regulating collagen expression depending on muscle contractile activity is at its very beginning. Elucidating the mechanism of function-dependent inhibition of collagen expression in interstitial fibrogenic cells is of prime importance. Regarding this issue, miR-206 function is of great interest. Increased expression and secretion of miR-206 (in the form of exovesicles) was recently shown to inhibit collagen expression in muscle fibroblasts present in the interstitial space between fibers [97]. Interestingly, a serum miR-206 level increases upon hindlimb suspension in mice [98]. Decreased collagen content during unloading can be possibly due to changes in this microRNA expression and transport. There is little information on miR-206 expression and vesicular secretion during gravitational unloading so far. Further research will elucidate the mechanisms regulating the collagen content in a muscle and its stiffness during unloading.

## THE ROLE OF SUPPORT AFFERENTATION IN MAINTAINING THE STIFFNESS PROPERTIES OF A POSTURAL MUSCLE

The direct effect of support afferentation on human motor functions was first shown in a joint Soviet-Cuban experiment aboard a Soviet spacecraft. Plantar mechanical stimulation was used in the experiment [99]. Modified devices were further used in dry immersion experiments, which enabled prolonged sessions of plantar stimulation. These studies revealed that support stimulation during immersion maintains a normal level of electrical activity and reflectory transverse stiffness in the soleus muscle [100].

The following protocol for plantar stimulation was used in our experiments: daily plantar pressure of 40 kPa. Stimulation was carried out for 6 h in total, with 20-min exposure sessions at the beginning of each hour using natural modes of locomotion: slow walking (75 steps/min) for 10 min and fast walking (120 steps/ min) for 10 min. No significant decrease in the CSA of slow-twitch muscle fiber and no noticeable change in the percentage ratio of fiber expressing slow- and fasttwitch isoforms of myosin heavy chains were noted in the soleus muscle after 7-day immersion using plantar stimulation [101]. Thus, atrophy was prevented without the use of intense running or resistive loads. The use of plantar stimulation prevented a decrease in the maximum isometric tension and the calcium sensitivity of permeabilized fiber [19, 101, 102]. The obtained results indicate that muscle activity induced by stimulation of support afferents makes it possible to avoid disruptions in cross-bridge formation.

The studies on the transverse stiffness of the myofibrillar apparatus (atomic force microscopy following pretreatment of permeabilized fiber with Triton X-100) using application of plantar stimulation during 7-day immersion demonstrated a significant decrease (by 30%) in stiffness only in the Z-disc plane in relaxed fiber. Transverse stiffness in all other sarcomere regions did not differ statistically significantly from the pre-immersion values [19]. The use of plantar stimulation did not completely prevent stiffness reduction in activated fibers (pCa, 4.2). However, the resulting stiffness drop varied within a range of 15%–25% in different sarcomere regions. Thus, the decrease in the activated fiber stiffness was significantly less pronounced after plantar stimulation compared to that after immersion alone [19]. Apparently, muscle activity enabled preservation of the stiffness of the myofibrillar apparatus by preventing both disruption in cross-bridge formation and breakdown of sarcomeric cytoskeletal proteins. The latter suggestion is supported by the data on the titin and nebulin contents in the human soleus muscle, which were obtained using plantar stimulation during dry immersion. The titin and nebulin contents in individuals in the group of plantar stimulation during dry immersion showed only a slight tendency to decrease, while the same parameters in the group with dry immersion only decreased by something like 40% [101, 102]. A reduced desmin content was not observed during plantar stimulation, either. Since a breakdown of the above cytoskeletal proteins is usually ascribed to the activity of μ-calpain, we may suggest that muscle activity induced by afferent stimulation initiates an endogenous mechanism of calpain inhibition. This mechanism may be associated with maintenance of a high activity of nitric oxide synthase, which is known as an endogenous inhibitor of calpain activity (see above). In our study, plantar mechanical stimulation not only prevented a decrease in the content of neuronal nitric oxide synthase, but also slightly increased its content compared to the pre-immersion level [103]. Further studies will show whether our suggestions about the mechanism underlying support afferentation are valid. These are the mechanisms by which support afferentation, providing a constant (albeit low) activity level in a postural soleus muscle, maintains the normal state of the cytoskeleton and actin–yosin motor mobilization system.

## STIFFNESS AND ATROPHY

Skeletal muscle stiffness is not only the mechanical basis for antigravitational stability in mammals, but also an integral component of the mechanotransduction system: i.e., the transformation of mechanical alteration of muscle fiber structures into a metabolic signal regulating gene expression, protein synthesis, and protein degradation. Over the years, numerous publications (e.g., [104]) have discussed a potential signaling role for titin. However, for a long time, there have been almost no experimental data to substantiate these suggestions. The only evidence of a signaling role for titin was translocation of E3 ubiquitin ligase MuRF2 bound to the kinase domain of the titin M-line region to the muscle nucleus during gastrocnemius muscle denervation [105]. In addition, the same research group reported increased ATPase activity and phosphorylation of the titin kinase domain upon titin stretching *in vitro *[106].

The following questions remain open. The first relates to how the titin kinase domain localized in the sarcomere M-line region and involved in dimerization of titin molecules bound to two adjacent myosin filaments can serve as a mechanosensor. The second question is about exactly what mechanical signal it perceives. It was hypothesized that this domain may serve as a sensor for disordering myosin filaments and that it is the sarcomere structure disruption that triggers sarcomeric protein synthesis [107]. This hypothesis is based on a mathematical model of sarcomere mechanics, which also takes into account the contribution of some extra-sarcomeric cytoskeletal proteins of the M-line (mainly obscurin). The suggestion on the involvement of obscurin in the stabilization of thick filaments in sarcomeres was further confirmed in experiments with the flight muscle of obscurin-knockdown Drosophila [108].

Recent experiments on hemidiaphragm denervation compared the signaling properties of muscles in two mutant mouse lines with either increased or decreased titin stiffness. Denervation atrophy was prevented by muscle mechanical stretching stimulating anabolic processes. The anabolic effect of stretching was found to be more pronounced in mice with increased titin stiffness [109]. According to this report, the anabolic signal was transmitted using a specific ankyrin repeat protein bound to titin. This protein was released from the complex with titin and entered muscle nuclei upon stretching. It is believed to stimulate the expression of the genes regulating anabolic processes in fiber. Thus, the mechanical signal of muscle stretching could transform into a chemical signal that further stimulated protein synthesis.

Based on numerous reports on the anabolic effect of stretching, as well as eccentric and resistive loading in general, a number of authors believe that the source of muscle atrophy during gravitational unloading is not the cessation of fiber contractile activity but rather a decreased tension, i.e. load capacity, resistance of muscle contraction [11, 110]. This conclusion is mainly supported by experiments with chronic low-frequency electrical stimulation combined with suspension [110 , 111, 112]. Even partial prevention of atrophy in the soleus muscle was not achieved in these experiments. Interestingly, the use of repeated electrical stimulation instead of continuous stimulation prevents not only muscle weight loss, but also a decrease in passive muscle stiffness [113, 114, 115]. We used 7-day immersion, combined with mechanostimulation of support afferents, and obtained a significant decrease in the muscle atrophy degree without creating additional tension in the soleus muscle [6, 101]. The use of plantar mechanostimulation in experiments with short-term (1–3-day) hindlimb suspension in rats fully prevents an elevation in proteolytic enzyme expression and partially prevents a decrease in the protein synthesis rate [116]. We may suggest that, at least at the initial stage of unloading, the contractile activity caused by activation of support afferents counteracts the breakdown of the rigid cytoskeletal network and overcomes its intrinsic resistance, thus allowing partial or complete prevention of muscle atrophy.

## CONCLUSION

Thus, the facts known to date indicate the following:

– Intrinsic muscle stiffness in human and rodent limbs, both transverse and longitudinal, as well as dynamic and static, naturally decreases as early as during the first week under support withdrawal; the most pronounced stiffness decrease is observed in the Z-disc zone;

– The decrease is accompanied by a reduction in the content of sarcomeric cytoskeletal proteins, both giant ones (titin and nebulin) and the Z-disc proteins stabilizing titin filaments; the contribution of changes in the nature of actin–myosin interactions to a stiffness decrease during gravitational unloading seems insignificant;

– Cytoskeletal proteins are degraded by calpains, members of the family of calcium-dependent cysteine proteases, which are regulated by nitric oxide synthase and some heat shock proteins;

– Activation of muscle contractions under support afferentation reduces the cytoskeletal protein breakdown rate and maintains the level of muscle stiffness close to its native level; and

– Intrinsic muscle stiffness and activity of cytoskeletal proteins are a prerequisite for preventing the atrophy of inactive muscles.

The current state of the issue of the molecular mechanisms reducing the passive stiffness of a postural muscle in simulated gravitational unloading leaves a number of important problems unresolved, which include:

– What sarcomeric component (cross-bridges, giant cytoskeletal proteins, as well as minor and regulatory proteins) changes are responsible for decreased stiffness in an isolated muscle at different time intervals of animal exposure to gravitational unloading?

– What processes lead to breakdown/inactivation of sarcomeric proteins during unloading?

– What role does a decrease in the intensity of defense mechanisms play in these processes?

– Does extracellular matrix proteins (mainly collagens) affect the processes of reducing isolated muscle stiffness?

– What are the mechanisms of cytoskeletal protein influence on the signaling pathways regulating anabolic processes in fiber, and does a decrease in muscle stiffness affect these mechanisms?

The search for answers to these questions could prove exhilarating for future research.

## Abbreviations

CSA: cross-sectional area
Akt: protein kinase B
GSK3β: glycogen synthase kinase 3β
HSP: heat shock protein
17-AAG: 17-(allylamino)-17-demethoxygeldanamycin
